# The Arabidopsis MTP8 transporter determines the localization of manganese and iron in seeds

**DOI:** 10.1038/s41598-017-11250-9

**Published:** 2017-09-08

**Authors:** Heng-Hsuan Chu, Suzana Car, Amanda L. Socha, Maria N. Hindt, Tracy Punshon, Mary Lou Guerinot

**Affiliations:** Department of Biological Sciences, Dartmouth College, 78 College Street, Hanover, NH 03755 USA

## Abstract

Understanding how seeds obtain and store nutrients is key to developing crops with higher agronomic and nutritional value. We have uncovered unique patterns of micronutrient localization in seeds using synchrotron X-ray fluorescence (SXRF). Although all four members of the *Arabidopsis thaliana* Mn-CDF family can transport Mn, here we show that only *mtp8-2* has an altered Mn distribution pattern in seeds. In an *mtp8-2* mutant, Mn no longer accumulates in hypocotyl cortex cells and sub-epidermal cells of the embryonic cotyledons, but rather accumulates with Fe in the cells surrounding the vasculature, a pattern previously shown to be determined by the vacuolar transporter VIT1. We also show that MTP8, unlike the other three Mn-CDF family members, can transport Fe and is responsible for localization of Fe to the same cells that store Mn. When both the VIT1 and MTP8 transporters are non-functional, there is no accumulation of Fe or Mn in specific cell types; rather these elements are distributed amongst all cell types in the seed. Disruption of the putative Fe binding sites in MTP8 resulted in loss of ability to transport Fe but did not affect the ability to transport Mn.

## Introduction

**Figure 1:**
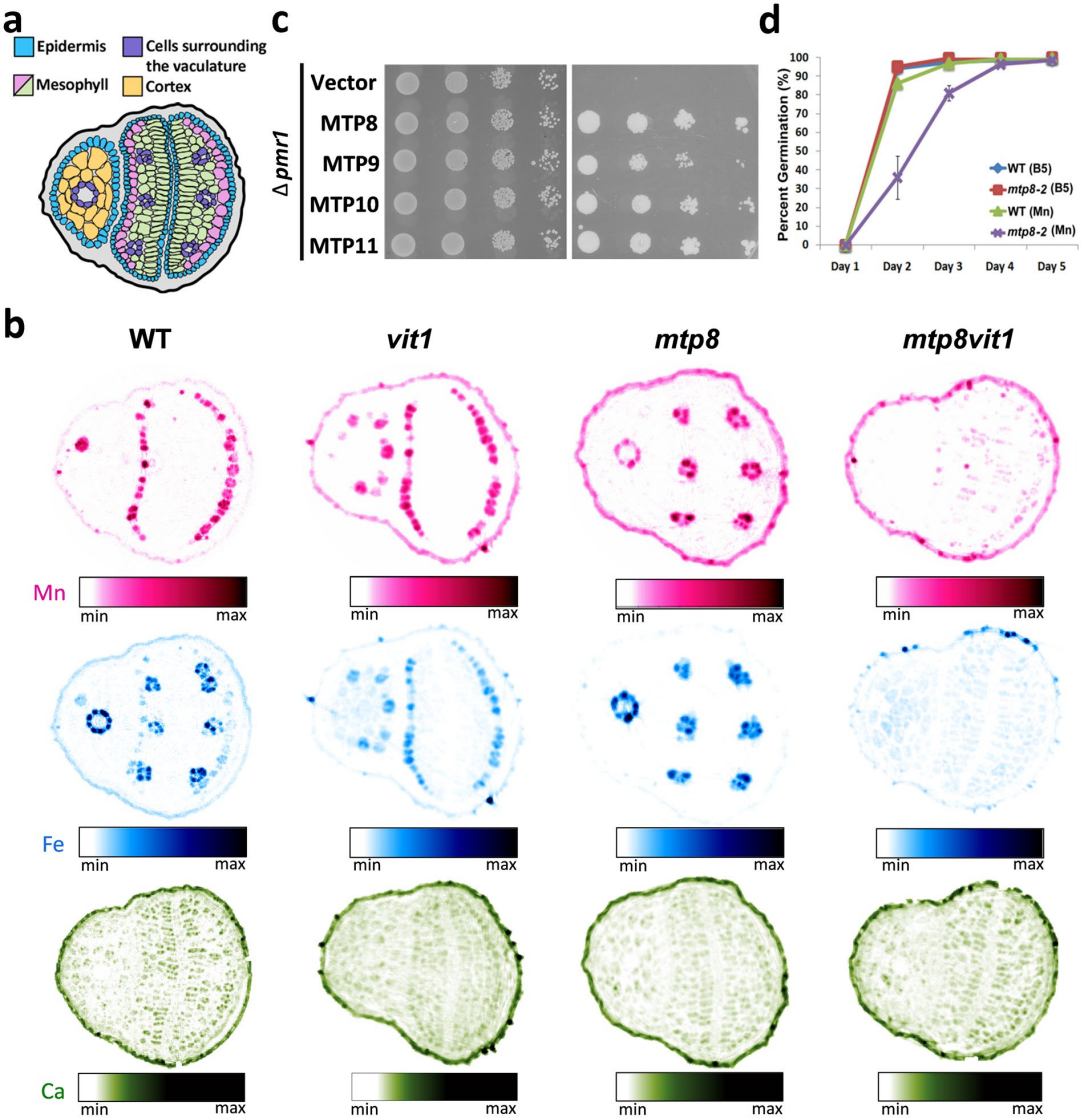
MTP8 is a Mn transporter involved in Mn distribution in seeds. (**a**) Schematic representation of a cross section through the middle of a mature Arabidopsis seed. (**b**) Loss of MTP8 and VIT1 changes Mn and Fe patterning in the seed. High resolution (1 μm) tomograms (virtual slices) through the center of WT, *vit1*, *mtp8-2* and *mtp8vit1* double mutant seeds show altered Fe and Mn distribution at the cellular level. Ca is found distributed throughout the seed and is unchanged in mutant seeds. Each image is individually processed and scaled to show the relative intensity of the element being mapped. As such, quantitative comparisons should not be made between images. (**c**) Yeast functional complementation. Cells of the Mn-hypersensitive yeast mutant Δ*pmr1* transformed with empty vector, MTP8, MTP9, MTP10 or MTP11 were grown on synthetic defined medium with ﻿(right panel) or without (left panel) 4 mM Mn. 7 μl of 10 fold serial dilutions were spotted onto the medium. (**d**) Percent germination of WT and *mtp8-2* on B5 medium with (Mn) or without (B5) supplementation of 1.5 mM Mn.

We are interested in seeds as sources of micronutrients due to the high prevalence of micronutrient deficiencies in many human populations that consume primarily plant-based diets^1, 2^. Nutrient-rich seeds also offer agronomic benefits such as increased seedling vigor, resistance to disease and other stresses, and increased crop yields^3^. SXRF has emerged as a powerful tool that provides spatial localization of elements in living plant tissues; as such, it can be used to understand nutrient loading in seeds. High-resolution SXRF tomograms have revealed unique patterns of micronutrient localization in seeds^4–8^, raising questions of how these patterns are set up, whether the patterns are biologically significant, and whether they can be altered in support of biofortification of staple crops^9–11^. In wild type *Arabidopsis thaliana* embryos, Fe predominantly localizes to the vacuoles of cells surrounding the vasculature in the radicle, hypocotyl and cotyledons (Fig. 1)^4, 12^. Mn localizes to cortical cells in the radicle and hypocotyl and to a single layer of spongy mesophyll cells on the abaxial side of the cotyledons. The vacuolar transporter VIT1 is responsible for the localization of Fe to the cells surrounding the vasculature^4^. In a *vit1* loss-of-function mutant, Fe no longer accumulates in these cells, but instead accumulates in the same cells as Mn. *vit1* seeds have the same concentration of Fe as wild type seeds. However, when Fe is limiting, *vit1* mutants die shortly after germination, suggesting proper localization of Fe to the cells surrounding the vasculature is essential for seedling survival^4^.

Although VIT1 can transport Mn in addition to Fe, the spatial pattern of Mn accumulation in *vit1* and wild type seeds is the same^4^. We hypothesized that there must be a vacuolar Mn transporter distinct from VIT1 that is responsible for determining the Mn pattern in seeds. Such a transporter could also play a role in the sequestration of Mn in other plant tissues^13^. Mn is an essential nutrient needed for myriad cellular processes, including photosynthesis, respiration, protein synthesis and hormone activation^14^. For example, Mn is a component of the oxygen-evolving complex in photosystem II that catalyzes the water splitting reaction to produce oxygen and provides electrons for the photosynthetic electron transport chain. As a constituent of MnSOD, Mn is also required in protection against reactive oxygen species (ROS). Like other metals, Mn can be toxic in excess^14^. Indeed, Mn toxicity is second only to Al toxicity as the most significant metal toxicity of plants grown in acidic soils (pH of 5.5 or lower). Approximately 30% of the world’s total land area is composed of acidic soils, and nearly 50% of the world’s potentially arable lands are acidic^15^. This is a major constraint to agricultural production worldwide.

Among the possible candidates for determining the Mn pattern in seeds are the Metal Tolerance Proteins (MTPs) that belong to the Cation Facilitator Family (CDF) in Arabidopsis. The CDFs are phylogenetically clustered into three subgroups, Zn-CDF, Fe/Zn CDF, and Mn-CDF^16–18^. The CDF proteins act as proton antiporters responsible for effluxing divalent cations from the cytoplasm to the outside of the cell or into subcellular compartments. As such, these transporters have been implicated in conferring metal tolerance; they can also allow metals to be safely stored for subsequent use. Among the twelve MTPs identified in *A. thaliana*, four (MTP8, MTP9, MTP10 and MTP11) are clustered into the Mn-CDF sub-family whose members share a consensus sequence DxxxD (Supplementary Fig. S1), that has been shown to be a Mn binding motif^19^; this motif is not found in the Zn- or Fe/Zn-CDFs^16^.

MTP11 was the first *A. thaliana* Mn-CDF to be functionally characterized^20, 21^. MTP11 confers tolerance to excess Mn when expressed in yeast. In Arabidopsis, it localizes to pre-vacuolar or Golgi-like compartments, suggesting that it confers Mn tolerance through vesicular trafficking and subsequent exocytosis of excess Mn^21^. *mtp11* mutants are hypersensitive to high Mn whereas overexpression of *MTP11* increases tolerance to high Mn^20, 21^. MTP11 orthologs in other species also localize to a Golgi-like compartment^21, 22^. MTP9 has been characterized in rice where it is mainly expressed in roots and is required for the translocation of Mn to the stele^23^. MTP9 is localized to the proximal side of the plasma membrane on exodermal and endodermal cells, opposite the Mn uptake transporter Nramp5, which is found on the distal side^23^.

ShMTP8 (previously annotated as ShMTP1) from the Mn hyperaccumulator *Stylosanthes hamata*, confers Mn tolerance by sequestering excess Mn in the vacuole^24^. The cucumber MTP8 ortholog can also confer Mn tolerance when expressed in Arabidopsis whereas a rice Tos-17 insertion line of MTP8 exhibited symptoms of Mn toxicity when grown under excess Mn conditions^25, 26^. Recently, expression of Arabidopsis MTP8 (AtMTP8) was shown to increase under both low-Fe and high-Mn growth conditions, and the *mtp8* mutant was only hypersensitive to Fe deficiency when Mn was present in the medium^27^. MTP8 is localized to the tonoplast and can complement a Mn-hypersensitive yeast strain^27^. Altogether, these results support MTP8 functioning as a vacuolar Mn transporter that protects plant cells from Mn toxicity under both excess Mn and Fe deficiency stresses^27^.

Here, we show that MTP8 is involved in Mn and Fe localization in seeds. We also show that MTP8, unlike the other three Mn-CDF family members, can transport Fe and is responsible for its localization to the same cells that store Mn. Disruption of the putative Fe binding sites in MTP8 resulted in loss of ability to transport Fe but did not affect the ability of the mutated MTP8 protein to transport Mn. *mtp8* loss-of-function mutant plants are sensitive to high Mn, while *MTP8* overexpression lines are more tolerant to high Mn, confirming that MTP8 plays a role in protecting plants from high levels of this essential, but potentially toxic metal, presumably by sequestering it into vacuoles.

## Results and Discussion

We first asked if all of the Arabidopsis Mn-CDF proteins could functionally complement the Mn-hypersensitive yeast mutant Δ*pmr1*. PMR1 is a P-type ATPase responsible for transporting Ca and Mn into the Golgi, a major pathway for cellular detoxification of Mn in yeast^28^. All of the yeast strains showed similar growth on synthetic defined medium (Fig. 1c, left panel). Growth of Δ*pmr1* in the presence of high levels of Mn was restored in strains expressing MTP8, MTP9, MTP10, and MTP11, but not in the strain expressing the empty vector (Fig. 1c, right panel), suggesting that all of the predicted Mn-CDFs in Arabidopsis are able to alleviate Mn toxicity by mobilizing excess Mn out of the yeast cytoplasm. This conclusion is further supported by the reports on other members of the Mn-CDF family^19–22, 25–27^.

Examination of publically available gene expression data revealed that *MTP8*, *MTP9*, *MTP10* and *MTP11* are all expressed in seeds (Supplementary Fig. S2). Of the four *A. thaliana* Mn-CDFs, MTP8 had the highest expression during embryogenesis, with expression increasing over the course of embryo development whereas MTP11 expression decreases during this same time period. To investigate whether any of the four MTPs were involved in the localization of Mn in seeds, we isolated homozygous T-DNA lines. All four lines are complete loss-of-function for their respective gene as no transcripts could be detected (Supplementary Fig. S3). We used SXRF 2D elemental mapping to visualize metal distributions in intact wild type and T-DNA mutant seeds (Supplementary Fig. S4). In wild type seeds, Fe (shown in blue) is localized to the cells surrounding the vasculature of the embryo, including that of the radicle, hypocotyl and embryonic cotyledons. Mn (shown in magenta) is localized to the abaxial side of the embryonic cotyledons [Supplementary Fig. S4 and as previously described^4, 12^]. *mtp9, mtp10*, and *mtp11* display the same metal localization pattern as wild type seeds (Supplementary Fig. S4). *mtp8-2*, however, has an altered Mn accumulation pattern. Mn is no longer localized to the abaxial side of the embryonic cotyledons; instead it co-localizes with Fe (Supplementary Fig. S4; areas of co-localization are shown in purple). In order to examine the distribution pattern more closely, we imaged virtual slices (tomograms) of wild type and *mtp8-2* seeds at 1 µm resolution. Tomograms provide spatial localization of metals without the need for sample embedding or sectioning, eliminating the risk of element contamination or redistribution during sample preparation. Our high-resolution microtomography analysis showed that Mn localizes to cortical cells of the hypocotyl and to sub-epidermal mesophyll cells on the underside of the cotyledons whereas Fe localizes to the cells surrounding the vasculature of the hypocotyl and cotyledons as we had previously reported (Fig. 1b and Supplementary Fig. S5). In the virtual slice shown for the wild type seed, only one cortical cell is seen to have accumulated Mn. We often see only one or two cortical cells that have accumulated Mn in any given section but upon reconstruction of the entire seed, it is evident there is no apparent pattern as to which cortical cells accumulate Mn. At present, we cannot offer an explanation for why some, but not all, cortical cells are accumulating Mn. In addition, imaging with higher resolution than was used in our previous work (4) shows that some Fe co-localizes with Mn in wild type seeds (Fig. 1b). Tomograms of *mtp8-2* mutant seeds show a striking difference in both Mn and Fe localization compared to wild type. Fe and Mn localize exclusively to the cells surrounding the vasculature of the hypocotyl and cotyledon; both metals are absent from the mesophyll and cortical cells of the cotyledon and hypocotyl, respectively (Fig. 1b and Supplementary Fig. S5). The distribution of other elements is unchanged in the *mtp8-2* mutant compared to wild type.

Colocalization of Mn and Fe in the *mtp8-2* mutant suggests that VIT1 is a good candidate for storing Mn in seeds when MTP8 is not functioning. To test our hypothesis, we generated an *mtp8vit1* double mutant and examined Mn and Fe localization in its seeds. In double mutant seeds, the unique patterns of Mn and Fe distribution are no longer evident, with both Mn and Fe dispersed throughout whole seed (Fig. 1b and Supplementary Fig. S5). This suggests that MTP8 is responsible for Fe storage when VIT1 is not functioning and that VIT1 is responsible for the storage of Mn when MTP8 is disrupted.

**Figure 2:**
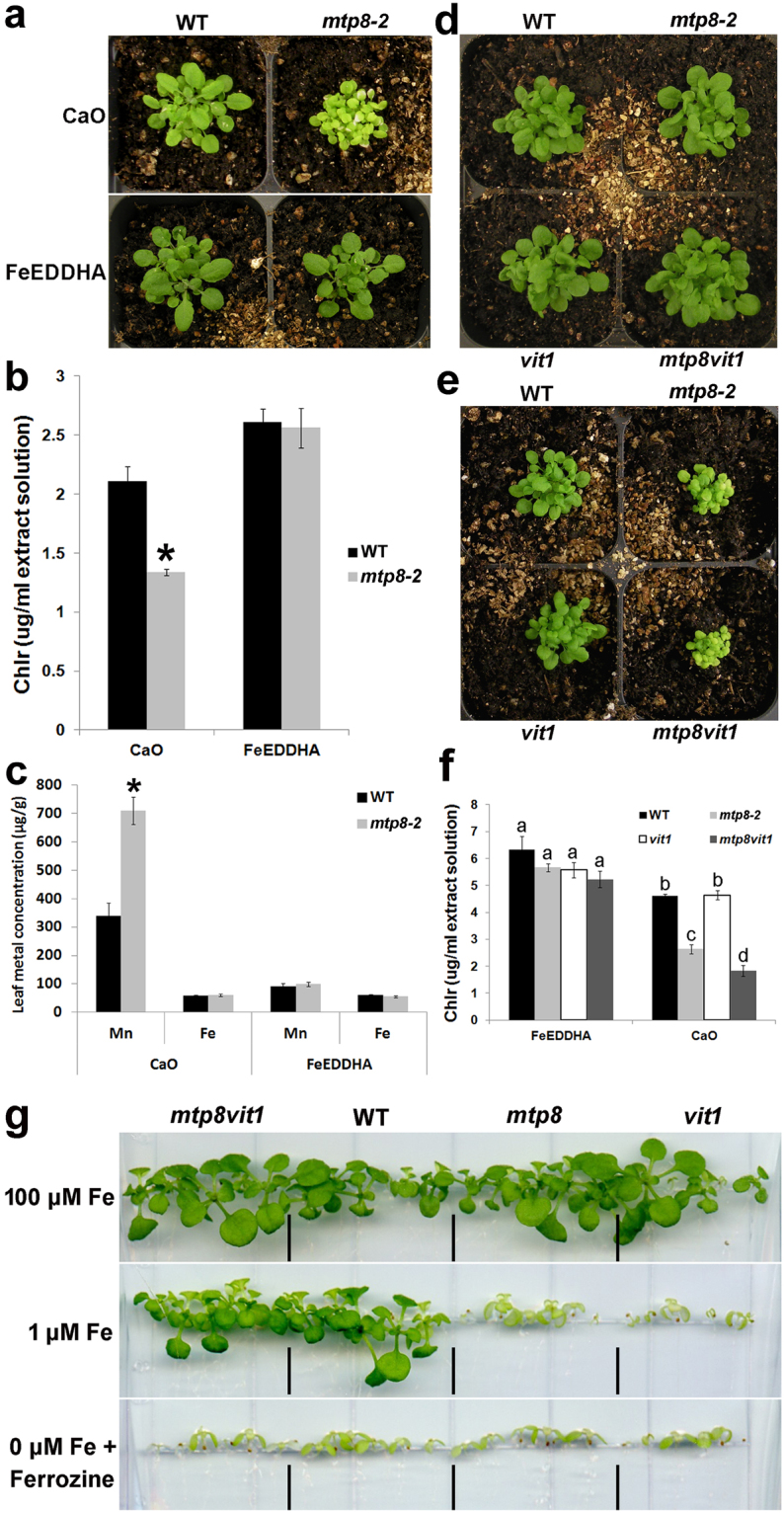
Growth of wild type (WT), *mtp8-2*, *vit1*, and *mtp8vit1* on alkaline soil and low Fe media. (**a**) WT and *mtp8-2* plants grown for 3 weeks on promix (soil with no starter nutrients) watered with CaO (1 g/L) or CaO and FeEDDHA (0.5 g/L) (FeEDDHA). (**b**) Chlorophyll levels of WT and *mtp8-2* plants in (**a**). (**c**) Mn and Fe concentrations of leaves of WT and *mtp8-2* mutant plants. Error bars represent SE (n = 8). Asterisks indicate P < 0.05 by *t* test. (**d**,**e**) WT, *mtp8-2*, *vit1*, and *mtp8vit1* plants grown for 3 weeks on promix watered with CaO (1 g/L) (**e**) or CaO and FeEDDHA (0.5 g/L) (FeEDDHA) (**d**). (**f**) Chlorophyll levels of WT and *mtp8-2* plants in (**d**,**e**). Columns marked with different letters represent significantly different means according to a statistical analysis (P < 0.05, ANOVA, Tukey’s test). (**g**) WT, *mtp8-2*, *vit1*, and *mtp8vit1* plants grown for 2 weeks on 100 μM Fe, 1 μM Fe and 0 μM supplemented with 300 μM Ferrozine.

After determining that MTP8 is necessary for the localization of Fe and Mn to sub-epidermal cells in the cotyledons as well as to cortical cells in the radicle/hypocotyl, we asked whether the altered localization of these metals in *mtp8-2* seeds influences seed germination and/or seedling vigor. When germinated on B5 plates, *mtp8-2* was indistinguishable from wild type (Fig. 1d). However, when grown on B5 supplemented with high levels of Mn, *mtp8-2* seeds exhibited delayed germination compared to wild type (Fig. 1d). When plants were grown on high Fe medium, on the other hand, germination of *mtp8-2* was similar to that of wild type (Supplementary Fig. S6). When we germinated *mtp8-2* seeds on low Mn and Fe medium, seedling germination was not affected. We also measured the elemental composition of *mtp8-2* seeds and compared it to that of wild type seeds. There was no significant change observed in Mn or Fe concentrations in *mtp8-2* seeds relative to wild type (Supplementary Fig. S7). A similar result was seen with the *vit1* loss-of-function mutant where the concentration of Fe was the same as in wild type despite the dramatic change in Fe localization^4^. Under our current growth conditions, *vit1* mutants did not have a dramatic seedling lethal phenotype when germinated in alkaline soil; they were, however, smaller than wild type (Fig. 2e). *mtp8-2* mutants also have a phenotype when grown in alkaline soil (Fig. 2a). They are smaller, have lower chlorophyll content (Fig. 2b) and have significantly higher concentrations of Mn in their leaves than wild type plants (Fig. 2c). All of these phenotypes can be reversed by the addition of FeEDDHA (Fig. 2a–c). It is likely that the inability to sequester Mn in the vacuole causes problems under low Fe conditions where plants are accumulating Mn and that these problems are alleviated by the addition of Fe, which downregulates IRT1, thus preventing the uptake of Mn via IRT1. IRT1 has previously been shown to transport Mn in addition to Fe^29^ and the *irt1-1* loss-of-function mutant fails to accumulate Mn under Fe-deficient conditions^30^. The reversal of *mtp8* phenotypes by the addition of FeEDDHA is consistent with the results reported by Eroglu *et al*.^27^, showing that *mtp8* mutants were only hypersensitive to Fe deficiency when Mn was present in the medium. Eroglu *et al*. also confirmed a previous report that *MTP8* expression is strongly induced under Fe deficiency in a FIT-dependent manner^31^; both our data and theirs support the conclusion that MTP8 is required for the detoxification of Mn under Fe deficiency conditions.

Since *mtp8vit1* showed completely altered Mn and Fe distribution compared to *mtp8-2* and *vit1* (Fig. 1b), we tested whether seed germination or seedling vigor was affected. The double mutant showed no germination defects and grew normally under Fe sufficient conditions (Fig. 2d). However, when grown in alkaline soil, the double mutant showed the same symptoms as *mtp8-2* when compared to wild type (Fig. 2e and f). Since *vit1* does not show a seedling lethality phenotype under this condition (Fig. 2e), the phenotype observed in the double mutant was presumably caused by an overaccumulation of Mn at the wrong location rather than by Fe deficiency. We also examined the double mutant on plates. No growth difference was observed when plants were grown under Fe sufficient conditions (Fig. 2g). Nevertheless, when grown on low Fe medium, both *mtp8-2* and *vit1* exhibit a seedling lethal phenotype while the *mtp8vit1* double mutant shows growth similar to that of wild type (Fig. 2g). We attribute the difference in growth of the double mutant on plates versus soil to differences in the concentrations of bioavailable Fe and Mn in plates versus soil. Cells need to maintain a certain Fe/Mn ratio. In the *mtp8* mutant, the inability to move Mn into the vacuoles causes Mn to accumulate in the cytosol of cells surrounding the vasculature, resulting in toxicity. In the *mtp8vit1* double mutant, both Fe and Mn are no longer stored in the vacuole of specific cells, but are evenly distributed in the cytosol of all cells and available for use. In conclusion, proper localization of Mn in the embryo is essential for seed germination and seedling growth under high Mn stress or Fe deficiency conditions.

**Figure 3:**
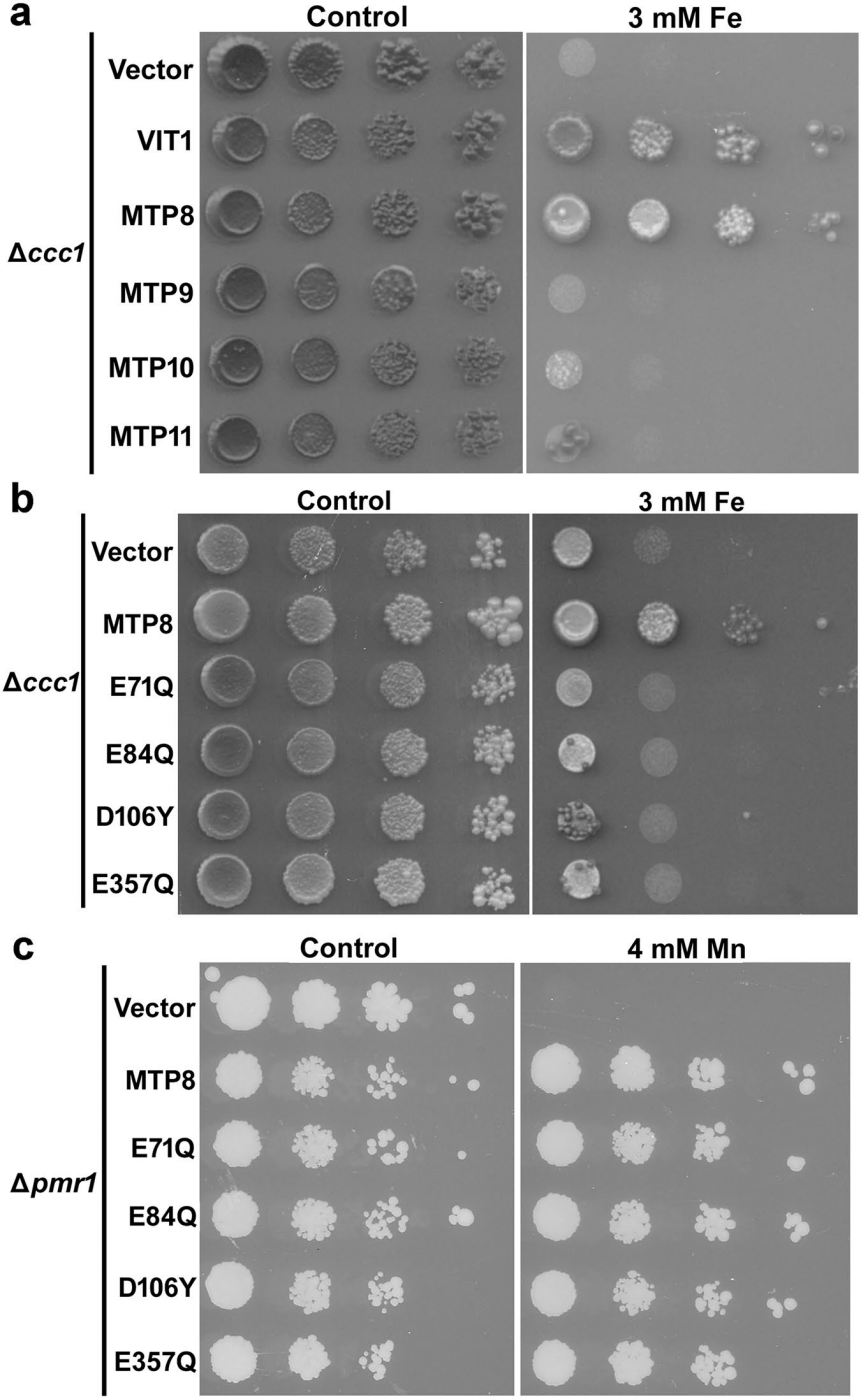
Yeast functional complementation. (**a**) The Fe-hypersensitive strain Δ*ccc1* transformed with empty vector, MTP8, MTP9, MTP10 or MTP11 were grown on synthetic defined medium with or without (Control) supplementation of 3 mM Fe. VIT1 was included as positive control. (**b**) The Fe-hypersensitive strain Δ*ccc1* transformed with empty vector, MTP8, MTP8.E71Q, MTP8.E84Q, MTP8.D106Y or MTP8.E357Q were grown on synthetic defined medium with or without (Control) supplementation of 3 mM Fe. (**c**) The Mn-hypersensitive strain Δ*pmr1* transformed with empty vector, MTP8, MTP8.E71Q, MTP8.E84Q, MTP8.D106Y or MTP8.E357Q were grown on synthetic defined medium with or without (Control) supplementation of 4 mM Mn. 7 μl of 10 fold serial dilutions were spotted onto the medium.

It is clear from the localization results that MTP8 must be able to transport Fe in addition to Mn. To confirm this, we tested whether MTP8 could complement the Fe hypersensitivity phenotype of the yeast Δ*ccc1* mutant. CCC1 encodes a vacuolar Fe transporter; inability of the Δ*ccc1* mutant to store Fe in the vacuole leads to an increased accumulation of cytosolic Fe and Fe hypersensitivity^32^. Figure 3a shows that only VIT1, the Arabidopsis ortholog of CCC1^4^, and MTP8 were able to restore growth of Δ*ccc1* under 3 mM Fe, suggesting that MTP8 is able to transport Fe into the vacuole whereas MTP9, MTP10 and MTP11 cannot.

MTP8 orthologs from *A. thaliana* (MTP8), *Stylosanthes hamata* (ShMTP8), *Oryza sativa* (OsMTP8.1) and *Cucumis sativus* (CsMTP8) share an E/DxxE/D Fe binding motif^33, 34^ which is not conserved in the other three *A. thaliana* Mn-CDFs (Supplementary Fig. S1). To examine whether the E/DxxE/D Fe binding motif contributes to the Fe transporter activity of MTP8, the four Fe binding motifs (Supplementary Fig. S1) were changed to Q/YxxE or ExxY using site directed mutagenesis; the function of the four mutagenized MTP8s, E71Q, E84Q, D106Y, and E357Q, were then examined for transport activity in Δ*ccc1*. E71Q, E84Q, D106Y, and E357Q were not able to restore growth of Δ*ccc1* (Fig. 3b) under 3 mM Fe, indicating that the mutations abolish Fe transport activity. This suggests that all four E/DxxE/D Fe binding motifs in MTP8 are required for transporting Fe from the cytosol into the vacuole. The Fe motif mutants were able to restore growth of Δ*pmr1* under 4 mM Mn (Fig. 3c) suggesting that the Mn transporter function of these mutagenized proteins is not disrupted by the point mutations and confirming that the mutant proteins are stable in yeast. Consistent with our results, Chen *et al*.^19^ showed that an N-terminal deletion does not affect the Mn transporter activity of OsMTP8.1. In addition, we showed that although the N terminus is not required for Mn transport, it is essential for Fe transporter activity (Supplementary Fig. S1).

**Figure 4:**
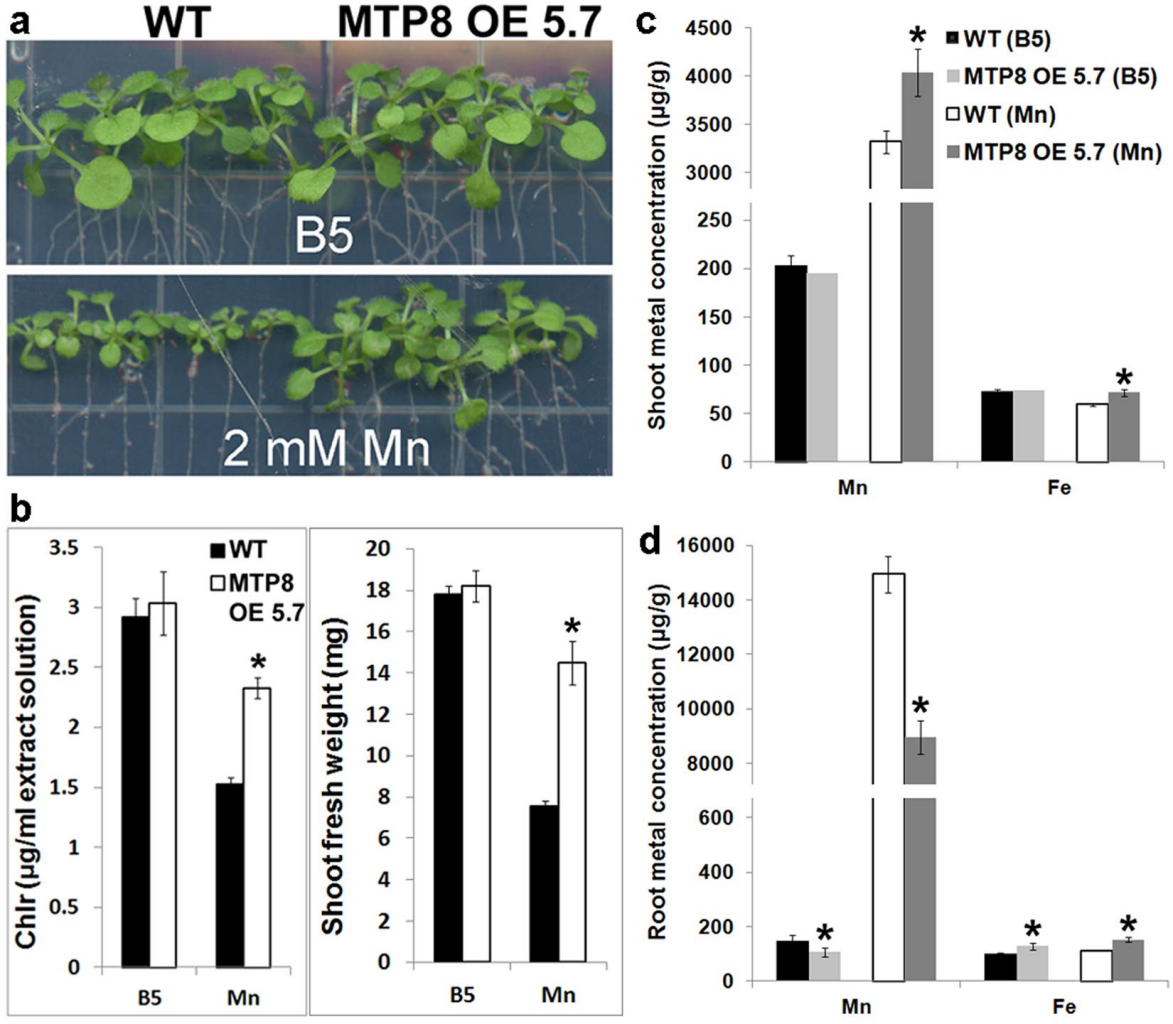
MTP8 OE plants are more tolerant to high levels of Mn. (**a**) Wild type (WT) and MTP8 OE plants grown on B5 (59 μM Mn) with or without supplementation of 2 mM Mn for 17 days. (**b**) Chlorophyll levels and shoot fresh weights of WT, and MTP8 OE plants in (**a**). Each biological replicate is a pool of 5 plants. Error bars represent SE (n = 3). Asterisks indicate P < 0.05 by *t* test. (**c**,**d**) Mn and Fe concentrations of shoot (**c**) and root (**d**) of wild type (WT) and MTP8 OE plants grown on B5 with or without supplementation of 1.5 mM Mn. Error bars represent SE (n = 5). Asterisks indicate P < 0.05 by *t* test.

As MTP8 is involved in localization of Mn and Fe in the seed, we wanted to know if overexpression of *MTP8* would lead to increased concentrations of Mn and/or Fe in seeds. Seeds of the line overexpressing *MTP8* had 13% more Mn than wild type seeds when grown on regular soil (Supplementary Fig. S8) and 60% more Mn than wild type seeds when grown on high Mn soil (Supplementary Fig. S8). Fe concentrations were the same as wild type. We also tested whether overexpression of *MTP8* could also enhance Mn tolerance of the plants as MTP8 has been reported to be important for Mn stress tolerance^27^. Lines overexpressing MTP8 show increased tolerance to excess Mn (Fig. 4a). Also, when grown on high Mn, the *MTP8* overexpression plants had significantly higher chlorophyll content and shoot fresh weight than wild type plants (Fig. 4b) despite the fact that their shoots contained significantly higher concentrations of Mn than the shoots of wild type plants (4039 ± 242 μg/g for MTP8 OE 5.7 versus 3320 ± 121 μg/g for wild type; Fig. 4c).

MTP8 localizes to the vacuole^27^. With the exception of Fe, which can be stored in the chloroplast as ferritin, heavy metals are mainly stored in the vacuole^13^. Although other transporters can import Mn and/or Fe into the vacuole such as CAX2, CAX4^35–37^ and VIT1^4^, MTP8 clearly plays a unique role in vacuolar import because disrupting MTP8 caused mislocalization of Mn and Fe in seeds relative to wild type. In addition to localization, MTP8 and VIT1 are primarily responsible for Mn and Fe storage in seeds respectively, and can function to create a alternative vacuolar sink when the other is not functional. Of course, the inability to retrieve Fe and Mn from the vacuole can also disrupt metal homeostasis, as clearly shown by studies of the AtNRAMP3 and AtNRAMP4 vacuolar transporters that are required for export of Fe into the cytosol during seed germination^38^ and for retrieval of Mn from the vacuole of mature leaves under Mn deficiency^39^. However, disruption of NRAMP3/4 does not alter the localization of Mn or Fe in seeds^40^. Our characterization of the MTP8 transporter has contributed to understanding how seeds store nutrients and how plants protect themselves from excess metals, both of which are key to developing crops with higher agronomic and nutritional value.

## Materials and Methods

### Plant materials and growth conditions

The Arabidopsis T-DNA insertion lines *mtp8-2* (SALK_140266), *mtp9* (SALK_093515), *mtp10* (SALK_121470 C), and *mtp11* (SALK_025271 C) were obtained from ABRC. Homozygous lines were identified by PCR using the left border T-DNA-specific and the gene-specific primers (Supplementary Table S1). Seeds were surface sterilized in 70% ethanol, 0.05% Triton X-100, and then imbibed in sterile distilled 0.1% agarose at 4 **°**C for 3–5 days. Plants were grown on full or half strength B5 medium (Sigma). MnSO_4_ was added from 100 mM stocks. For soil-grown plants, seeds were imbibed in sterile distilled 0.1% agarose at 4 **°**C for 3–5 days, and then sown directly onto autoclaved potting mix (MetroMix 820). Growth conditions for both plate-grown and soil-grown plants were 16 hour light, 8 hour dark at 22 **°**C.

### Yeast functional complementation

*Saccharomyces cerevisiae* strain BY4743Δ*pmr1 (pmr1::KANMX)* ^28^ and *DY150*Δ*ccc1 (ccc1::HIS3)* ^32^ were transformed with pAG426-EGFP (Addgene plasmid 14203) or pAG426GAL-EGFP carrying *VIT1*, *MTP8*, *MTP9*, *MTP10*, and *MTP11* cDNA, and amplified by PCR using indicated primer pairs (Table S1). For complementation assays, SD –Ura medium was made with 2% galactose supplemented with various concentrations of MnSO_4_ or FeSO_4_. Yeast suspensions were prepared from 3-day-old yeast colonies. Colonies were removed from the plates, suspended in sterile water, and the OD600 of the resulting suspension was measured. The OD600 of the suspension was brought to 1, serial dilutions (1:10, 1:100, 1:1000, and 1:10,000) of the suspension were then prepared and 7 µL of each dilution was spotted on the plates.

### Chlorophyll measurement

The shoots were harvested and assayed for chlorophyll content as previously described^41^.

### Plasmid construction

In the overexpression construct 35 S:*MTP8*, the MTP8 CDS was amplified using Phusion DNA polymerase (NEB) using indicated primer pairs (Table S1), and then cloned into pEarleyGate103^42^.

### Elemental analysis

Samples were dried at 60 °C for 3 days. Elemental analysis was done by ICP-MS as described^43^.

### Synchrotron X-Ray fluorescence computed microtomography

Intact seeds of Col, *mtp8-2, mtp9, mtp10*, and *mtp11* were analyzed for metal distribution using SXRF at beamline × 27 A of the National Synchrotron Light Source, Brookhaven National Laboratory. Seeds were mounted in the same orientation onto metal-free Kapton tape and positioned at a 45° angle to the incident beam. The Canberra High Purity Geranium array detector was used to measure X-ray fluorescence from the sample, positioned 90° from the incident beam. An incident energy of 11 keV was used to simultaneously excite the elements of interest. The step size used for the Col and *mtp8-2* images was 5 μm; for *mtp9, mtp10*, and *mtp11* images the step size was 10 μm. The dwell time for all images was 100 ms.

X-ray fluorescence (XRF) microtomography of Arabidopsis seeds was also conducted at the GSECARS hard X-ray microprobe beamline 13-ID-E (Argonne, IL). The 13-ID-E source is a 2.1 m long, 36 mm period undulator in a canted straight section that provides a tunable energy range between 2.4 and 28 keV. Monochromatic radiation was provided from a liquid nitrogen-cooled double crystal monochromator using a Si(111) crystal set. Incident beam energy for this experiment was tuned to 11 keV. A secondary source aperture collimated the monochromatic beam and a pair of 200 mm long, Rh-coated Si mirrors in a Kirkpatrick-Baez geometry were used to focus the beam to ~1 × 2 µm resolution (V x H). The measured incident photon flux for this experiment was ~ 4 × 10^10^ photons/sec. The incident flux was reduced to maximize elemental sensitivity while minimizing potential beam damage to the sample. XRF energy dispersive spectra were measured using a 4-element Si drift diode detector (Vortex-ME4, Hitachi High-Technologies Science America, Inc.) coupled to Quantum Xpress 3 digital spectrometers. Tomographic XRF analysis was conducted in air, in continuous scanning mode with the rotation axis (θ) configured as the fast (continuous) scan direction. Sinograms were collected using 360° (θ) rotation with full energy dispersive spectra from the XRF detector being binned every 0.5° in 20 ms and then translated 1 µm horizontally (X). The array size varied depending on the particular sample, but generally this provided megapixel tomograms on the order of 750 angles by 400 X steps. Tomographic reconstructions were done using software provided by GSECARS written in IDL (v. 7) and a filtered backprojection algorithm. Collecting sinograms over a full 360° of rotation minimized the effects of self-absorption for low atomic number elements (particularly Ca and K K_α_ emission lines) in the tomographic reconstruction. No other corrections were made to correct for self-absorption.

Arabidopsis seeds were mounted with the micropyle uppermost on quartz capillary tubing using Devcon™ 5-minute Epoxy resin as a metal-free adhesive. These were then mounted on a goniometer for tomographic analysis at the beamline. Tomograms were routinely collected through the midpoint of the seed. This configuration, particularly incorporating rapid flyscanning with rotation as the fast axis (which provided exceptional sample stability in scanning), allowed the collection of high resolution tomograms in just under two hours with reconstructed voxels of ~1 µm.

## Electronic supplementary material

Supplementary Information
