# Supplementary material for: The Arabidopsis MTP8 transporter determines the localization of manganese and iron in seeds
Source: Sci Rep. 2017 Sep 8;7:11024. doi: 10.1038/s41598-017-11250-9 (PMC5591227; doi:10.1038/s41598-017-11250-9)
Supplement: Supplementary file 1 — Supplementary Information [file 41598_2017_11250_MOESM1_ESM.pdf]

Supplementary Information

The Arabidopsis MTP8 transporter determines the localization of manganese and iron in seeds

Heng-Hsuan Chu, Suzana Car, Amanda L. Socha, Maria N. Hindt, Tracy Punshon and Mary Lou Guerinot

```
AtMTP8     -------------------------------------MEVNYCPETPLLSS--------   14
CsMTP8     ---------------------------------MDGDS--DLSPKAPLL-------GAS   17
OsMTP8.1   -------------------------------------------------------MEAKG   5
ShMTP8     ---------------------------------MDANSGSDPNIKRPLLSMHHASSAAS   26
AtMTP11    ------------------------------MVE---PASPDSDEGIS--LLEF--------   18
AtMTP9     ------------------------------MAATEHRGLSGGDYNVD--LLPID-------   22
AtMTP10    MPLNSYIFFLFLTTSPRNTFFGIRTHSDRIMATEHITRTGDEYNVE--LLPSD-------   51

AtMTP8     -NDHEAIDHKPKLTGMVSSMKSNFFADLPQKLRSKI-DPENPLHLDVSKAAGLKEDEKEY   72
CsMTP8     LNGSGGKRGRLSRRYSVNSLRSEFISRLPDKLRSHLQDVESPYEIDLSKSSGFSREEKDY   77
OsMTP8.1   ENDARAPLLAERRRNSVGSMRGEFVSRLPKKVLDAV-DPERPSHVDFSRSKGLREGEKEY   64
ShMTP8     ENGTR--RSPLKRRNSVNSLRSAFLAKIPDKVRASL-DSESLSNLDLSDSTALTPGEKEY   83
AtMTP11    -----HGNGDRSWQLNFDD----FQVSPEHKE----KKSPS-KLHNCLGCLGPEDNVADY   64
AtMTP9     ---QDDSP-PSSWRLSLDT----FRLPSSSPLSSGRHNGRT-RLSRYLRTPKKERKVSEY   73
AtMTP10    ---DDAPPLESSWRLNLDA----FQLPSS---TGGRHDGRT-RFSRYFRTPRKERRVSEY   100
                           ..     *             .                    :*

                                                               I
AtMTP8     YERQLATLKSFEEVESFLARSDEYTIDEKEEEEDRAERAAQELAMQISNWANIFLLALKI   132
CsMTP8     YERQLATLKSFEDVDSLVSSDC------I-DEEDMEEGAQQERAMKISNYANIVLLLLKI   130
OsMTP8.1   YEKQFATLRSFEEVDSIEESNV------MSEEDDIAEQKQSEFAMKISNYANMILLALKI   118
ShMTP8     YEKQIATLKSFEEVDAIVDRDT------VIDDADDEEQRQQERAMRISNYANVVLLILKI   137
AtMTP11    YQQQVEMLEGFTEMDELAERGFVPGM--SKE-EQDNLAKSETLAIRISNIANMLLFAAKV   121
AtMTP9     YKQQEKLLEGFNEMETINETGFVSGA--PTEEELKKLAKSERLAVHISNAANLVLFVAKV   131
AtMTP10    YKKQERLLEGFNEMETIHENGFASGV--PTEEEMKKLAKSERLAVHISNATNLVLFVAKV   158
           *::*   *..* ::: :   .         :         .  *::*** :*:.*:  *:

                              II
AtMTP8     YATVKSGSIAIAASTLDSLLDLMAGGILWFTHLSMKNVNIYKYPIGKLRVQPVGIIIFAA   192
CsMTP8     YATVRSGSIAIAASTLDSLLDLMAGGILWFTHLYMKQVNIYKYPIGKLRVQPVGIIVFAA   190
OsMTP8.1   YATIKSGSIAIAASTLDSLLDLMAGGILWFTHLSMKSINVYKYPIGKLRVQPVGIIIFAA   178
ShMTP8     YATVRSGSLAIAASTLDSLLDLMAGGILWFTHLSMKNINIYKYPIGKLRVQPVGIIIFAA   197
AtMTP11    YASVTSGSLAIIASTLDSLLDLLSGFILWFTAFSMQTPNPYQYPIGKKRMQPLGILVFAS   181
AtMTP9     YASVESRSMAVIASTLDSLLDLLSGFILWFTANAMRTPNNFRYPIGKRRMQPVGIIVFAS   191
AtMTP10    YASMESRSMAVIASTLDSLLDLLSGFILWFTANAMRKPNQFHYPIGKRRMQPVGIIVFAS   218
           **:: * *:*: **********::* *****   *:  * ::***** *:**:**::**:

            III                                      IV
AtMTP8     VMATLGFQVLLVAAEQLISNEPSEKMNHVQLIWLYSIMLSATAIKLVLWIYCKSSRNHIV   252
CsMTP8     VMATLGFQVLLQAVEQLIQDKPSESLSSEQFVWLCAIMTFATVVKLALWLYCKNSRNDIV   250
OsMTP8.1   VMATLGFQVFVQAVEKLIVNETPDKLTPVQLTWLYSIMIFATVVKLALWLYCRTSGNKIV   238
ShMTP8     VMATLGFQVLITALEELIQNSPAERMTQEQLIWLYSIMIFATVVKLCLWLYCRTSRNQIV   257
AtMTP11    VMATLGLQIILESLRTMLSSHKEFNLTKEQESWVVGIMLSVTLVKLLLVLYCRSFTNEIV   241
AtMTP9     VMATLGLQVILESTRLLVSK-NGSHMSSTEEKWMIGIMASATVVKFLLMLYCRSFQNEIV   250
AtMTP10    VMATLGLQVLLESGRQLVAK-SGIHMNSTEEKWMIGIMVSVTIVKFLLMLYCRGFQNEIV   277
           ******:*::: : . :: .     :.  :  *: .**  .* :*: * :**:   *.**

                               V                           VI
AtMTP8     RAYAKDHHFDVVTNVLGLVAAVLANAFYWWLDPTGAILLAIYTIVNWSGTVMENAVSLIG   312
CsMTP8     RAYAKDHYFDVVTNVVGLVAAILGDKIFWWIDPVGAIALAIYTILNWSGTVWENAVSLVG   310
OsMTP8.1   RAYAKDHYFDVVTNVVGLAAAVLGDMFYWWIDPVGAIALAVYTITNWSGTVWENAVSLVG   298
ShMTP8     RAYADDHHFDVVTNVVGLVAAVLGDKFYWWIDPIGAILLAVYTITNWSRTVMENAVSLVG   317
AtMTP11    KAYAQDHFFDVITNIIGLIAVILANYIDYWIDPVGAIILALYTIRTWSMTVLENVNSLVG   301
AtMTP9     RAYAQDHLFDVITNSVGLATAVLAVKFYWWIDPSGAILIALYTISTWARTVLENVHSLIG   310
AtMTP10    RAYAQDHLFDVVTNSIGLATAVLAVKFYWWIDPTGAILIALYTIATWARTVLENVHSLIG   337
           :***.** ***:** :** :.:*. : :*:** *** :*:*** .*: **  **. **:*

AtMTP8     QSAPPEVLQKLTYLVMRQGGDNIKHVDTVRAYTFGVLYFVEVDIELPEDLPLKEAHAIGE   372
CsMTP8     KSAPPEVLQMLTYLVIR-H-PEVKRVDTVRAYTFGVLYFVEVDIELPEELPLKEAHAIGE   368
OsMTP8.1   ESAPPEMLQKLTYLAIRHH-PQIKRVDTVRAYTFGVLYFVEVDIELPEELPLKEAHAIGE   357
ShMTP8     QSAPPEFLQKLTYLVVR-H-PQVKRIDTVRAYTFGVLYFVEVDIELPEELPLKEAHAIGE   375
AtMTP11    KSARPEYLQKLTYLCWNHH-KAIRHIDTVRAYTFGSHYFVEVDIVLPADMPLQVAHDIGE   360
AtMTP9     RSAPPDFLAKLTFLIWNHH-EKIKHIDTVRAYTFGSHYFVEVDIVLPEDMRLHEAHNIGE   369
AtMTP10    RSAPPDFLAKLTFLIWNHH-EQIKHIDTVRAYTFGSHYFVEVDIVLPEDMRLQEAHNIGE   396
           .** *: *  **:*  .     ::::*********  ******* **  :: *: ** ***

AtMTP8     SLQIKLEELPEVERAFVHLDFECHHKPEHSVLSTIPNDL-   411
CsMTP8     TLQIKIEKLPEVERAFVHLDFECEHKPEHSILSRLPNTQP   408
OsMTP8.1   SLQIKIEELPEVERAFVHLDFECDHKPEHNILSKLPSSQP   397
ShMTP8     TLQIKLEKLPEVERAFVHLDFECDHKPEHSVLVKLPNNQS   415
AtMTP11    SLQEKLELLEEIERAFVHLDYEYTHKPEHARSHC------   394
AtMTP9     TLQEKLEQLSEVERAFVHIDFEFTHRPEHKCKV-------   402
AtMTP10    TLQEKLEQLAEVERAFVHIDFEFTHRPEHKCN--------   428
           :** *:* * *:******:*:*  *:***
```

Supplementary Fig. S1. Alignment of MTP proteins. The alignment was carried out using Clustal Omega (1-3) and includes MTPs from *Arabidopsis thaliana* (AtMTP8-11), *Stylosanthes hamata* (ShMTP8), *Oryza sativa* (OsMTP8.1), and *Cucumis sativus* (CsMTP8). Black boxes indicate the conserved Mn binding motif DxxxD found in all Mn-CDFs. Lines above the sequence indicate the transmembrane domains. Conserved D/ExxD/E Fe binding motifs are shown in red, and red dots indicate AtMTP8 amino acids that were mutagenized (see Fig. 3b). An * (asterisk) indicates positions which have a single, fully conserved residue. A : (colon) indicates conservation between groups of strongly similar properties - scoring > 0.5 in the Gonnet PAM 250 matrix (4). A . (period) indicates conservation between groups of weakly similar properties - scoring =< 0.5 in the Gonnet PAM 250 matrix.

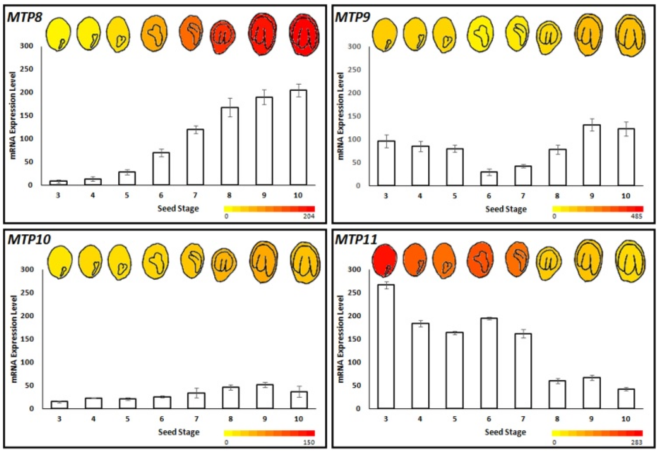

Supplementary Fig. S2. Expression Levels of *MTP8*, *MTP9*, *MTP10*, and *MTP11* in seeds. Shown are the mRNA expression levels of *MTP8*, *MTP9*, *MTP10*, and *MTP11* in different stages of seed development. The developmental stages included are 3 through 10 (5). Seeds are color coded based on a yellow to red color gradient, where yellow represents no expression and red is the absolute expression maximum for a particular gene. The figure was modified from the eFP Browser (6).

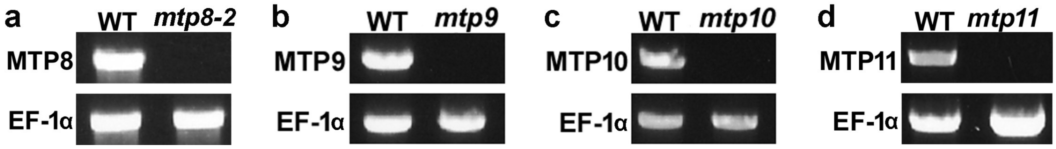

Supplementary Fig. S3. Semi-quantitative RT-PCR of plants grown on B5 for 10 days. Amplification of EF-1α is included as control. (a) *MTP8* mRNA level in WT and *mtp8-2*. (b) *MTP9* mRNA level in WT and *mtp9*. (c) *MTP10* mRNA level in WT and *mtp10*. (d) *MTP11* mRNA level in WT and *mtp11*.

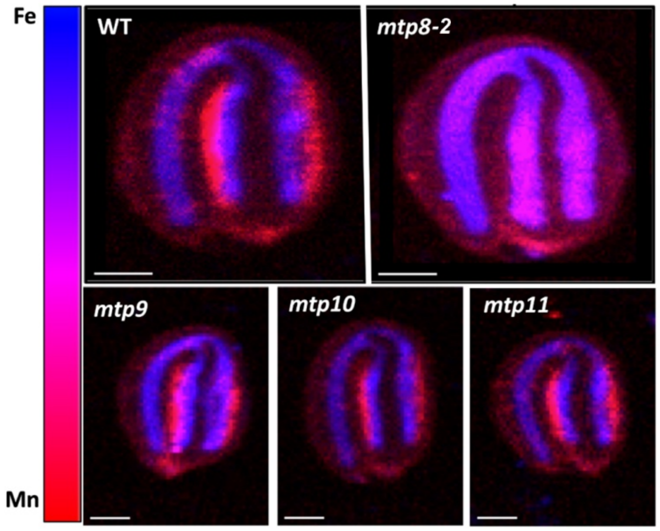

Supplementary Fig.S4. Loss of MTP8 results in altered seed Mn distribution. 2D mapping using Synchrotron X-ray imaging shows Mn distribution in wild-type (WT), *mtp8-2*, *mtp9*, *mtp10*, and *mtp11* seeds. WT and *mtp8-2* at 5 µM resolution and *mtp9*, *mtp10*, and *mtp11* at 10 µM resolution. Bar = 100 µM. Each image is individually scaled to show the relative intensity of the element being mapped. As such, quantitative comparisons should not be made between images.

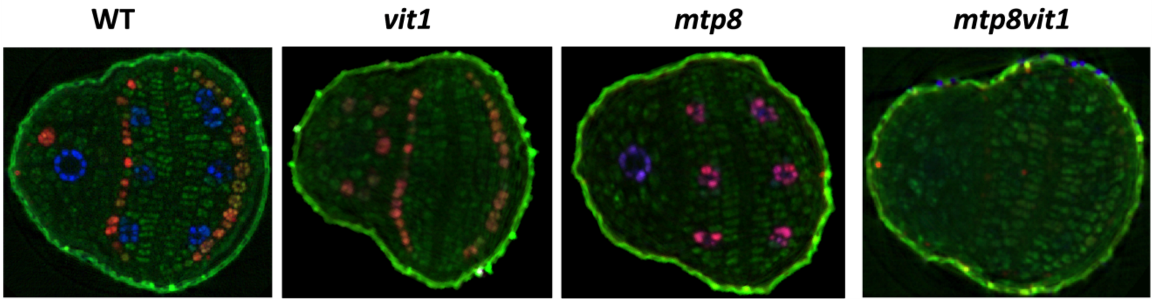

Supplementary Fig. S5. Overlays of the high resolution tomograms shown in Figure 1. These virtual slices through the center of WT, *vit1*, *mtp8* the *mtp8vit1* double mutant seeds show that loss of MTP8 and VIT1 changes Mn and Fe patterning in the seed. Each image is individually processed and scaled to show the relative intensity of the element being mapped, so quantitative comparisons should not be made between images. Red is Mn; Blue is Fe; Green is Ca.

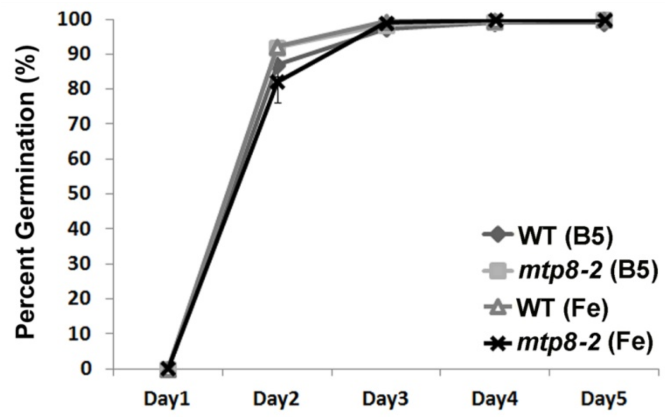

Supplementary Fig. S6. Percent germination of WT and *mtp8-2* on half strength B5 (50 μM Fe) with (Fe) or without (B5) supplementation of 500 μM Fe.

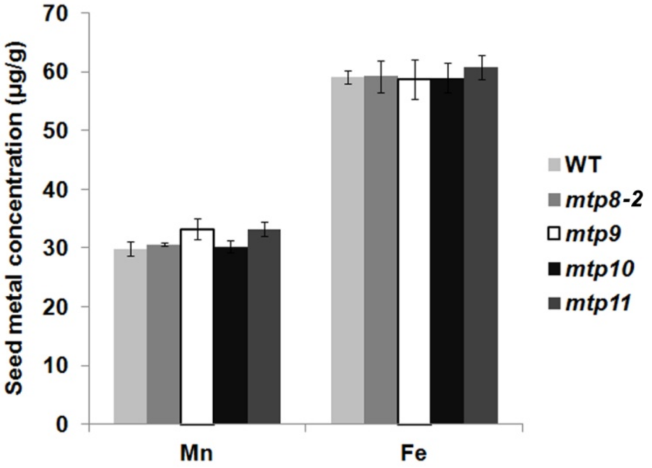

Supplementary Fig. S7. Mn and Fe concentrations of seeds of wild-type (WT), *mtp8-2*, *mtp9*, *mtp10*, and *mtp11*. Error bars represent SE (n=5). Asterisks indicate P < 0.05 by *t* test.

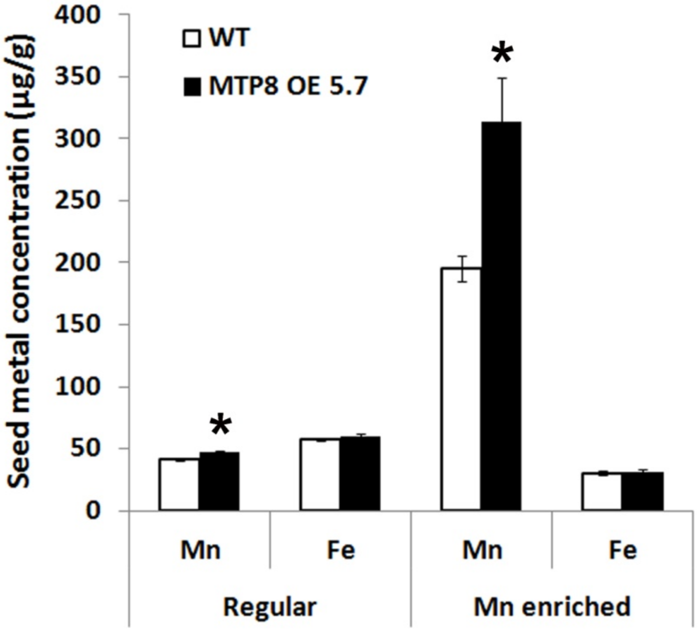

Supplementary Fig. S8. Mn and Fe concentrations of seeds of wild-type (WT) and the overexpression line (MTP8 OX 5.7) grown on regular soil watered with (Mn enriched) or without (regular) 1 mM MnSO4 once per week. Error bars represent SE (n=8). Asterisks indicate $P < 0.05$ by *t* test.

Supplemental Table 1. List of primers.

Name	Sequence	Purpose
LBb1.3	ATTTTGCCGATTTCGGAAC	genotyping
AtMTP8c.Fw	ATGGAAGTCAATTATTGTCCGGAAAC	cloning of CDS
AtMTP8c.RevSC	TCCTAAATCGTTGGGGATTGTAGAAAG	genotyping, cloning of CDS and genomic DNA
AtMTP8c.731-748	CCGCTGTTATGGCTACCC	genotyping
AtMTP8p.Fw	GATTTTCAAAGGATTCACCAAGTTTATAACC	cloning of genomic DNA
AtMTP8.qPCR Fw	TACCGTCCGTGCATATACCTTCG	Real-time PCR
AtMTP8.qPCR Rev	TGGCAGTTCTATATCCACCTCGAC	Real-time PCR
AtMTP8c.g211c Fw	GCCTCAAAGAAGATGAAAAGCAGTACTACGAAAGACAATTG	cloning of MP8.E71Q
AtMTP8c.g211c Rev	CAATTGTCTTTCGTAGTACTGCTTTTCATCTTCTTTGAGGC	cloning of MP8.E71Q
AtMTP8c.g250c Fw	AATTGGCAACACTAAAATCCTTTCAAGAAGTAGAAAGTTTCTTAGC	cloning of MP8.E84Q
AtMTP8c.g250c Rev	GCTAAGAAACTTTCTACTTCTTGAAAGGATTTTAGTGTTGCCAATT	cloning of MP8.E84Q
AtMTP8c.g316c Fw	CCATCGATGAAAAAGAAGAAGAAGAATATCGAGCTGAGAGA	cloning of MP8.D106Y
AtMTP8c.g316c Rev	TCTCTCAGCTCGATATTCTTCTTCTTCTTTTTCATCGATGG	cloning of MP8.D106Y
AtMTP8c.g1069c Fw	CTTTATTTTGTCGAGGTGGATATACAACTGCCAGAGGA	cloning of MP8.E357Q
AtMTP8c.g1069c Rev	TCCTCTGGCAGTTGTATATCCACCTCGACAAAATAAAG	cloning of MP8.E357Q
AtMTP9c.Fw1	CTCGTAACTTCCCCCACAAAATCA	cloning of CDS
AtMTP9c.RevSC	TCCAACCTTGCATTTGTGTTCT	cloning of CDS
AtMTP9.501-530	ATATGTTAACCCTGTGAAAATTGTAGAGTG	genotyping
AtMTP9.1146-1126	AAGGTTTGCTGCGTTTGAGAT	genotyping
AtMTP10c.Fw	ATGCCGCTTAACTCCTATATTTTCTTTC	cloning of CDS
AtMTP10c.Rev-SC	CTtGTTACACTTGTGTTCAGGACG	genotyping, cloning of CDS
AtMTP10.1578-1602	GCTTTATTGCAGAGGATTTCAGAAC	genotyping
AtMTP11c.Fw	ATGGTTGAGCCAGCTAGTC	cloning of CDS
AtMTP11c.RevSC	CTAACAGTGGGATCTAGCGTGC	cloning of CDS
AtVIT1c.Fw	CGATCATCAACAATGTCGTCGGAGGAAGA	cloning of CDS
AtVIT1c.RecSC	CTTATGTTGCACAACTTTAGCCAAAC	cloning of CDS

Supplementary References

1. Goujon, M., *et al.* (2010) A new bioinformatics analysis tools framework at EMBL-EBI. *Nucleic Acids Res.* 38:W695-699.
2. Sievers, F., *et al.* (2011) Fast, scalable generation of high-quality protein multiple sequence alignments using Clustal Omega. *Mol. Syst. Biol.* 7:539.
3. McWilliam, H., *et al.* (2013) Analysis tool web services from the EMBL-EBI. *Nucleic Acid Res.* 41:W597-600.
4. Dayhoff, M. O., Schwartz, R. M., & Orcutt, B. C. (1978) A model of evolutionary change in proteins. *Atlas of Protein Sequence and Structure*, ed Dayhoff M. O. (National Biomedical Research Foundation, Silver Spring, MD), Vol 5, pp 345-358.
5. Schmid, M., *et al.* (2005) A gene expression map of Arabidopsis development. *Nature Genet.* 37:501-506.
6. Winter, D., *et al.* (2007) An "Electronic Fluorescent Pictograph" browser for exploring and analyzing large-scale biological data sets. *PLoS ONE* 2:e718.
